# A Case of Severe Fioricet Withdrawal Presenting During Admission to an Inpatient Psychiatric Unit

**DOI:** 10.1155/2021/6371953

**Published:** 2021-11-08

**Authors:** Alejandro Rodulfo, Alberto Augsten, Erin Wainwright, Gil Abramovici

**Affiliations:** Memorial Healthcare System, Hollywood, Florida, USA

## Abstract

**Background:**

Butalbital-containing combination (BCC) analgesics have the potential for the development of tolerance and dependence. Misuse and withdrawal of these agents should be considered in patients presenting with new-onset psychosis. This case highlights how butalbital withdrawal may be missed in the emergency department setting and underscores how early identification may affect management and prognosis. *Case Presentation*. A 40-year-old female with a history of migraine, depression, and anxiety presented to the emergency department (ED) with new-onset psychosis following a recent seizure-like episode. At home, the patient was prescribed butalbital-acetaminophen-caffeine (Fioricet), duloxetine, alprazolam, and zolpidem for these conditions. On arrival to the ED, the patient was disoriented and appeared to be responding to internal stimuli. Following initial medical evaluation, the patient was cleared for further psychiatric assessment, during which she developed acute-onset autonomic instability while waiting for a bed on the inpatient psychiatric unit. She then became agitated, requiring multiple emergency medications, and eventually required emergent intubation and was admitted to the intensive care unit (ICU). Following extubation, a psychiatric consultation was performed. On assessment, the patient was alert and oriented and no longer exhibited psychotic symptoms. She admitted to using butalbital-acetaminophen-caffeine (Fioricet) daily for the past 10 years and had recently run out of her prescribed medication. She acknowledged that she was taking more than prescribed and requested substance use treatment resources, for which she was subsequently discharged to an inpatient drug rehabilitation facility.

**Conclusions:**

Given the time constraints inherent to the ED setting, a complete substance use history (both illicit and prescribed) may be challenging to obtain. However, it remains critical for providers to identify patients at risk for life-threatening withdrawal from sedative, hypnotics, and anxiolytic agents.

## 1. Introduction

Introduced in the 1970s, butalbital-containing combination (BCC) analgesics have been widely prescribed off-label for abortive treatment of migraine headaches. Butalbital, a short-acting barbiturate, has the potential to cause psychological dependence, physiological tolerance, addiction, and severe withdrawal. Reports on BCC overuse and withdrawal suggest that butalbital increases the risk of developing a substance use disorder, especially with regular use. As such, the United Kingdom, France, Germany, and Australia have removed BCC from their markets [1]. Despite rising safety concerns, these agents continue to be routinely prescribed in the United States by emergency medicine and primary care providers [1].

Up to 12% of the general population suffers from migraine headaches. A US survey conducted in 2006 found that among 506 patients suffering from chronic migraine, 13.5% of them used BCC in the past year. The average frequency of use within this population was 15.9 days per month [2]. To date, there is scarce literature supporting the regular use of BCC for migraine management or suggesting a clinically significant enhancement in analgesia with butalbital. The role of BCC in the management of headaches and their intrinsic abuse potential have not been systematically addressed; therefore, there is insufficient information available to guide clinicians [3].

According to the DSM-5, the diagnosis of “sedative, hypnotic, and anxiolytic use disorder” is based on the following criteria: ongoing use despite negative personal consequences, occupational and interpersonal impairment, use in hazardous situations, tolerance, withdrawal syndrome and ongoing use to avoid it, using more or for a longer period than intended, significant time investment to obtain or use, stopping or reducing important activities in order to use, persistent desire to cut down use, and craving or strong desire to use [4].

A study performed by Friedman and DiSerio demonstrated that BCC provides significantly more relief of psychological tension and symptoms of anxiety associated with tension headaches than either acetaminophen/codeine or placebo [5]. It relieves one dimension of headache impact, termed affective distress [6]. BCC decreases affective distress in susceptible individuals, such as those suffering from axis I and II psychiatric diagnoses, especially those with a history of substance use disorder. This population is therefore at risk of increasing BCC use in times of elevated stress, creating the potential for misuse and dependence that clinicians should be cognizant of [7].

## 2. Case Presentation

A 40-year-old female with a history of migraine headaches, depression, and anxiety presented to the ED with altered mental status. Symptoms began six days following a new-onset tonic-clonic seizure for which the patient was admitted to an outside facility, and no cause for this seizure was determined. Over the preceding three days, she developed difficulty sleeping and bizarre behavior and seemed to be talking to herself and her affect had been labile (laughing and crying for no apparent reason). Additionally, she described Lilliputian hallucinations in the form of seeing small inanimate objects. These symptoms were paroxysmal, and concomitantly she presented with slurred speech and gait abnormalities resulting in falls. The patient had a twenty-year history of depression and anxiety, with no prior psychiatric hospitalizations. Additionally, she suffered from chronic migraine headaches over the past 10 years. She was prescribed as needed butalbital-acetaminophen-caffeine (Fioricet) 50 mg-325 mg-40 mg daily, duloxetine 40 mg daily, alprazolam 0.25 mg twice daily, and zolpidem 10 mg nightly for headaches, anxiety, and insomnia, respectively. Family denied any known history of substance abuse.

On initial evaluation by the emergency medicine physician, the patient appeared in no apparent distress. She was alert but oriented only to person and location. A mild, bilateral hand resting tremor was noted. Her thought content was remarkable for grandiose and paranoid delusions, and she denied suicidal and homicidal ideation. She did not endorse any overt perceptual disturbance but appeared to be responding to internal stimuli. Vital signs, basic laboratory, and imaging results were unremarkable. She was subsequently placed on an involuntary psychiatric hold and transferred to the psychiatric ED for evaluation and management upon medical clearance. Due to her reported seizure history and home prescription for benzodiazepines, a diazepam taper was initiated to prevent benzodiazepine withdrawal seizures.

A few hours after transfer to the psychiatric ED, the patient became increasingly bizarre, physically agitated, and tachycardic while waiting for a bed on the inpatient psychiatric unit. Treatment with one dose of intramuscular haloperidol 5 mg, lorazepam 2 mg, and diphenhydramine 50 mg was ineffective in treating the agitation, which continued to escalate. A “rapid response” code was activated, and she was transferred back to the medical ED for further medical evaluation.

In the ED, repeat workup including serum chemistry, blood counts, chest X-ray, and urine and serum urine toxicology was unremarkable. The patient developed autonomic instability with a heart rate of 137 beats per minute and a blood pressure of 121/108 mmHg. Due to persistent agitation, she received two doses of intravenous lorazepam 2 mg each, a dose of intramuscular haloperidol 5 mg, and a dose of intramuscular ziprasidone 10 mg over the course of 75 minutes. The patient subsequently required endotracheal intubation and mechanical ventilation for airway protection due to the risk of respiratory failure from multiple antipsychotic and sedative agents. She was transferred to the ICU for further management and was maintained on midazolam and propofol infusions for sedation and intravenous diazepam for suspected benzodiazepine withdrawal, the preliminary diagnosis at the time.

Several attempts were made to extubate the patient over the next two days, but these were unsuccessful due to intermittent agitation. On her third hospital day, she was extubated and psychiatry was reconsulted. On evaluation, she appeared alert, fully oriented, and in no apparent distress. She admitted to misusing her prescribed Fioricet and endorsing taking up to 10-15 tablets daily (500-750 mg of butalbital) in the context of significant psychosocial stressors over the preceding six-month period. She agreed to treatment for substance use disorder and was discharged directly to an inpatient drug rehabilitation facility and was referred to outpatient neurology for migraine management.

Given her presentation of recent seizures, new-onset psychosis, and autonomic instability in the setting of Fioricet abuse, the patient was diagnosed with barbiturate withdrawal syndrome.

## 3. Discussion

Sedative-hypnotic drugs include barbiturates and benzodiazepines. These agents have multiple clinical indications ranging from outpatient management of anxiety and insomnia to procedural anesthesia and seizure cessation [8]. Barbiturates function by binding to the Gamma-Aminobutyric Acid_A_ (GABA_A_) receptor subtype in the central nervous system (CNS). GABA is a major inhibitory neurotransmitter that binds to a ligand-gated chloride channel, thus increasing the length of time the channel remains open. As such, in the presence of bound GABA, greater amounts of negatively charged chloride ions enter the cell, hyperpolarizing the membrane. This ultimately inhibits action potentials resulting in CNS depression. At high concentrations, barbiturates have GABAergic properties, activating receptors independently of GABA binding. Barbiturates' depressant effects are enhanced through their additional ability to depress the action of glutamate, an excitatory neurotransmitter, on *α*-amino-3-hydroxy-5-methyl-4-isoxazolepropionic acid (AMPA) receptors. The dual mechanisms of action and the resultant deep central nervous system depression make barbiturates capable of inhibiting and preventing seizure activity and producing full surgical anesthesia [8]. Like barbiturates, benzodiazepines also bind to the GABA_A_ receptor, but they do so at a separate binding site, and they instead produce more frequent chloride channel opening. Their binding is dependent upon simultaneous binding of GABA leading to a decreased risk for respiratory depression compared to barbiturates, and benzodiazepines have become the preferred drugs for sedative-hypnotic indications [9].

Fioricet combines butalbital 50 mg, acetaminophen 300 mg, and caffeine 40 mg meant for the treatment of tension headaches. Typical dosing is 1-2 capsules every four hours as needed, with a maximum dose not exceeding six capsules per day. According to the package insert, it is recommended to limit the use of Fioricet to three or fewer days per month to avoid developing medication overuse headaches; this risk has been shown to increase significantly if used five or more days per month [10]. BCC are effective for acute headache relief, and when employed to treat more frequently occurring migraine headaches or medication overuse headaches, a compulsive pattern of barbiturate use can develop. Psychological dependence is likely to occur first, followed by physiologic dependence and tolerance that may develop over the course of weeks to months [11].

Withdrawal from barbiturates is similar in many respects to withdrawal from ethanol. Both are typified by a hyperkinetic and hypersympathetic period of delirium with hallucinosis followed by rapid resolution. It is proposed that removal of GABAergic inhibitory tone within the CNS is responsible for the hypertension, tachycardia, diaphoresis, tremors, hyperthermia, and seizures observed during withdrawal. Withdrawal occurs most frequently and severely with short- to intermediate-acting barbiturates, such as butalbital [8]. Delirium and convulsions may begin within 16 hours following the last ingestion and last up to five days, and most symptoms gradually decline over the course of two weeks [10].

Previous cases reported by Romero et al. [12] and Raja et al. [13] of severe butalbital withdrawal were reported in patients taking daily doses of ranging from 450 to 1000 mg daily. Notably, these case reports also showed patients presenting with prominent psychiatric symptoms and limited laboratory or diagnostic imaging findings. Additionally, these patients exhibited minimal improvement following administration of benzodiazepines and antipsychotics [12]. These patients showed rapid resolution of symptoms following the administration of phenobarbital, with cognition returning to baseline during four to seven days.

Phenobarbital may be employed as the first-line treatment for seizure cessation if barbiturate withdrawal is suspected. Phenobarbital can also be used for maintenance therapy until additional seizure etiologies are ruled out. If a patient is using a BCC, discontinuation should be performed judiciously to avoid rebound headaches and withdrawal seizures. There are several proposed discontinuation protocols following the general principles of slowly tapering off the short-acting medication, transitioning to a longer-acting barbiturate if necessary, and addressing underlying headache etiology [14].

To prevent recurrence of butalbital use, once stabilized, patients should be referred to neurology for assessment and treatment. Psychiatric comorbidities should be taken into consideration as compulsive behaviors, self-medication, or dysfunctional coping mechanisms may require additional attention; in these cases, supervised discontinuation may be indicated. Some patients can meet the criteria for referral to substance abuse treatment modalities as well [14].

To our knowledge, there are less than ten prior reported cases of butalbital withdrawal in the existing literature, with this case representing the first one in most recent years. Despite the trend towards a lower prescription rate for barbiturates in the outpatient setting, BCC continue to be commonly prescribed in the United States as abortive treatment for different headache subtypes; hence, it is important that providers should remain vigilant for this diagnostic possibility when assessing patients in the ED and obtaining a substance use history.

## 4. Conclusion

BCC are effective at providing acute relief from tension headaches that are resistant to other treatments. However, their narrow therapeutic index and addiction potential can be clinically troublesome. Patients that take BCC for headaches, especially those with comorbid psychiatric illnesses, are at increased risk of developing dependence and tolerance to these agents, which can lead to a life-threatening withdrawal syndrome if stopped abruptly.

Barbiturate withdrawal can be potentially missed or misdiagnosed, especially in patients taking other medications such as benzodiazepines, which can lead to a similar withdrawal syndrome. New-onset seizures, psychotic symptoms, and autonomic changes should raise concern for barbiturate withdrawal in patients with chronic headaches; the lack of initial response to conventional treatments for benzodiazepines or ethanol withdrawal should prompt reconsideration of the diagnosis and possible initiation of treatment for barbiturate withdrawal.

Medication reconciliations, including cross-referencing prescription databases, are an essential portion of patient history taking. Furthermore, clinicians should be aware that a negative urine toxicology screen for barbiturates may herald imminent withdrawal. Facilities should develop protocols detailing appropriate monitoring and treatment for suspected barbiturate withdrawal. Additionally, protocols should be developed to safely wean patients off these agents.

This case underscores that patients prescribed with BCC should be monitored closely as outpatients and the minimum effective dose necessary to avoid the potential for significant dependence or withdrawal should be prescribed. If headaches are more frequent than a few days per month or there is a history of substance abuse, a referral to a neurologist is recommended to determine an appropriate headache management strategy to avoid daily use of barbiturates. Additional substance use treatment and referral to a psychiatrist should also be considered if there are concerns for misuse.

## Data Availability

All information is located on the patient's electronic medical record of Memorial Healthcare System.
